# Gas–Solid Two-Phase Flow Pattern Identification Based on Artificial Neural Network and Electrostatic Sensor Array

**DOI:** 10.3390/s18103522

**Published:** 2018-10-18

**Authors:** Fei-fei Fu, Jian Li

**Affiliations:** 1 School of Physics and Technology, University of Jinan, Jinan 250022, China; 2 Key Laboratory of Energy Thermal Conversion and Control of Ministry of Education, School of Energy and Environment, Southeast University, Nanjing 210096, China; lijian.5401@seu.edu.cn

**Keywords:** electrostatic sensor array, artificial neural network, gas‒solid two-phase flow, flow pattern identification

## Abstract

A method for gas–solid two-phase flow pattern identification in horizontal pneumatic conveying pipelines is proposed based on an electrostatic sensor array (ESA) and artificial neural network (ANN). The ESA contains eight identical arc shaped electrodes. Numerical simulation is conducted to discuss the contributions of the electrostatic signals to the flow patterns according to the error recognition rate, and the results show that the amplitudes of the output signals from each electrode of the ESA can give important information on the particle distribution and further infer the flow patterns. In experiments, the average values and standard deviations of the eight output signals’ amplitudes are respectively extracted as the inputs of the ANN to identify four kinds of flow patterns in a pneumatic conveying pipeline, which are fully suspended flow, stratified flow, dune flow and slug flow. Results show that for any one of those two input values, the correct rates of the ANN model are all 100%.

## 1. Introduction

Flow pattern identification of gas–solid two-phase flow in pneumatic conveying pipelines is significant for the optimized design and operation of a pneumatic conveying system. Many researchers have reported on process tomography for the visualization of dielectric material distributions in conveying pipeline. For example, electrical capacitance tomography (ECT), as one of the most established process tomography techniques for imaging solid material distributions in conveying pipeline, has been widely used to identify flow patterns of gas‒solid two-phase flow in industrial processes [1,2,3]. Alternatively, artificial intelligence is a promising technology in terms of flow pattern identification for multiphase flow. Rosa et al. [4] used artificial neural networks (ANN) to classify six flow patterns of gas‒liquid flow. They used four statistical momentums and the probability density function of the average void fraction as ANN inputs. Mustafa et al. [5] used an ANN for the identification of gas‒liquid flow patterns. Before building the ANN model, there is a pre-processing stage using natural logarithmic normalization. It helps to reduce overlaps between flow patterns. Ghose et al. [6] used a conductivity probe to sample the signal in that instrument and an ANN to identify the flow patterns of the gas‒liquid flow.

A review of the literature relating to flow pattern identification based on ANNs, provides little information that could be used as a guide to flow pattern identification of gas‒solid two-phase flow in pneumatic conveying systems. Therefore, this work focuses on flow pattern identification of gas‒solid two-phase flow in a pneumatic conveying pipeline based on an ANN. It is difficult to extract the inputs of an ANN with the most useful contribution to flow patterns from the fluctuation signals of the gas‒solid two-phase flow, due to various types of flow patterns and many complicated factors influencing the form of flow patterns in pneumatic conveying pipelines. Thus, in this work, we also specify the reliability of the inputs of an ANN for representing flow patterns.

For the gas‒solid two-phase flow, charges are generated on particles by particle collisions. Accordingly, particle charging of the gas‒solid two-phase flow could contain lots of information regarding particle dynamics and particle properties [7,8,9,10]. In recent years, electrostatic sensor array (ESA) as a novel type of electrostatic sensor has been applied to detect particle concentration distribution in a cross section of pneumatic conveying pipeline, for that the sensing field of ESA is quite localized [11,12,13]. Furthermore, the concentration distribution of charged particles over the cross section of the conveying pipeline has a significant relationship with the gas‒solid flow patterns. Therefore, in our research, we adopt an ESA and would specify the ANN inputs form the output signals of the ESA by computational modelling.

This paper is structured as follows: in Section 2, the structure, working principle and computation modeling of the ESA, and ANN model are presented. In Section 3, the setup of the pneumatic conveying system is presented. Section 4 describes the flow patterns in the pneumatic conveying system and presents the identification results.

## 2. ESA and ANN

### 2.1. Structure and Working Principle of ESA

**Figure 1:**
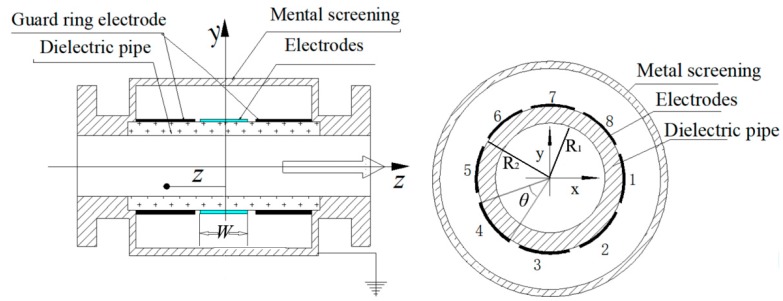
Schematic diagram of the ESA (*R*_1_ = 5 mm, *R*_2_ = 10 mm, *W* = 10 mm, *θ* = 40°).

The ESA is illustrated in Figure 1. Eight red copper sheets held tightly against the dielectric quartz glass tube are eight independent electrodes. A metal screening and two guard ring electrodes are used to resist electromagnetic interference. The pipe’s inside radius is denoted by *R*_1_, the outside radius by *R*_2_, the width of the electrode by *W* and the electrode angle by *θ*. Because of electrostatic induction, charges would be induced on each electrode of the ESA when charged particles flow through the testing pipeline.

### 2.2. Computational Modelling of ESA

**Figure 2:**
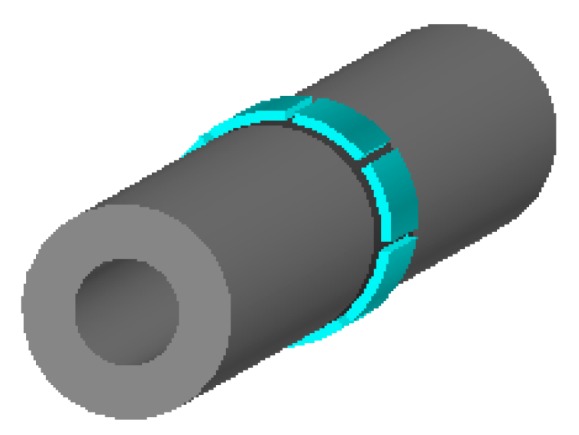
3-D structure model of electrodes set in Ansoft.

In this section, the feature values from the output signals of the ESA that contribute most to the gas‒solid flow patterns are analyzed using computational modelling. The computational model of the induced charge on each electrode has been established using the software Ansoft. Figure 2 shows a three-dimensional model of the electrodes set in Ansoft. The blue parts are eight electrodes and the grey part is a quartz glass tube.

**Figure 3:**
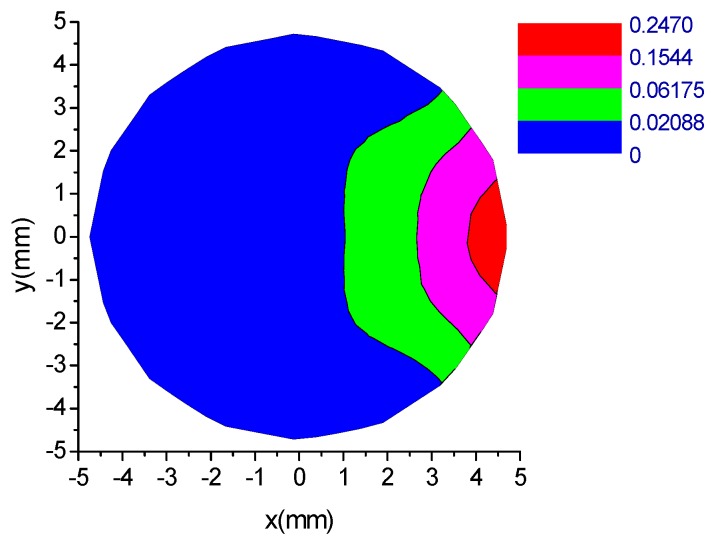
Sensitivity field of an electrode over the central cross section (*z* = 0) [12].

In earlier work, the sensitivity space of each electrode over the cross section (*z* = 0) of the test pipe was simulated [12]. Sensitivity is defined as the induced charge on an electrode when a unity point charge is positioned at different space coordinates in the sensing zone of the ESA [11]. The simulation result of the sensitivity characteristic of an electrode is shown in Figure 3. Each electrode has its own sensing space, and the sensing spaces of the eight electrodes are exactly alike due to axial symmetric distribution. It can be seen that the sensing space of an electrode is fan-shaped in the cross section (*z* = 0) of the test pipe and the sensing field is quite localized. The sensitivity decreases with the increase of distance from the electrode. In the red area the sensitivity is larger, and thus, the charged particles flowing near the electrodes would make the most significant contributions to the induced charge on corresponding electrodes.

**Figure 4:**
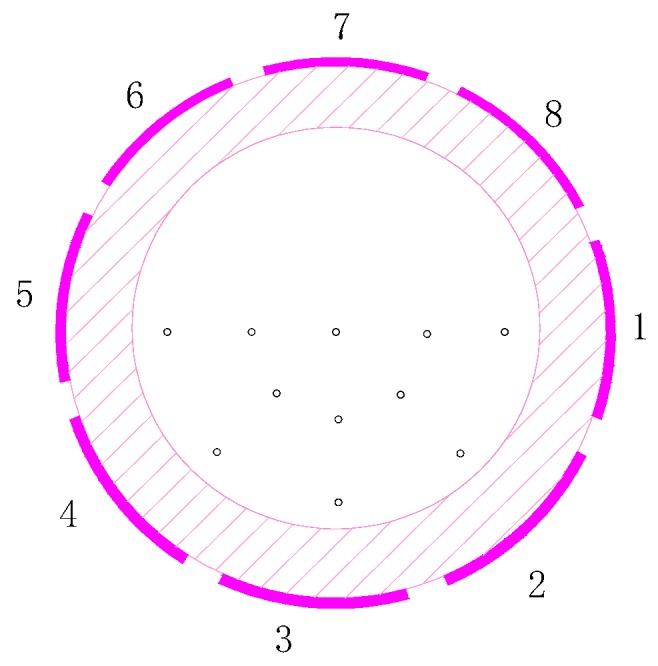
Distribution of the charged particles in the cross section of the pipe.

**Figure 5:**
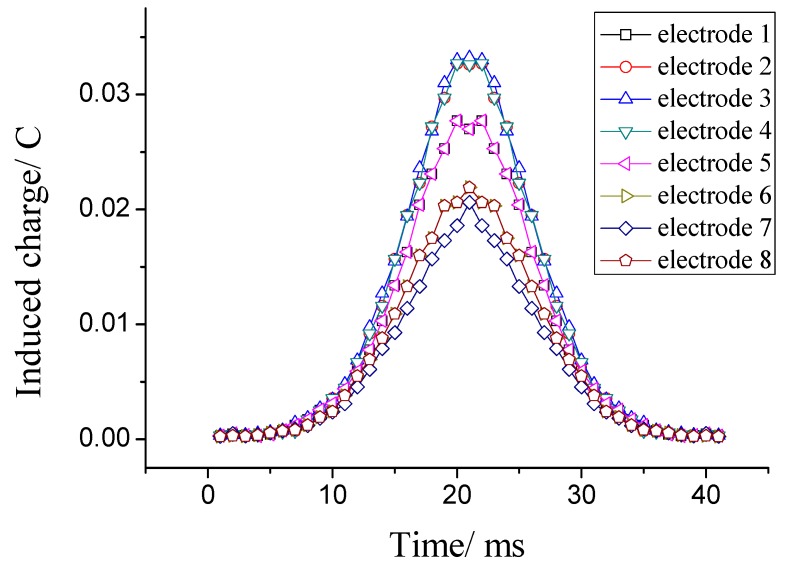
Induced charge on eight electrodes.

Secondly, the induced charges on the eight electrodes due to multiple charged particles are calculated based on sensitivity modelling. Particles in Ansoft are little balls with a diameter of 0.1 mm. Charges carried by each particle can be set through the parameter settings. Each particle carries a unity charge. Eleven particles move in undisturbed parallel lines (as shown in Figure 4). Other relevant parameters of these particles include the same velocity of 1 m/s, same axial position at any time, and move distance in *z* direction of −20 mm~20 mm. After the charged particles’ parameters have been set, the induced charges on each electrode can be calculated. Simulation results are shown in Figure 5. It can be seen that there are four types of induced charge curves. The curves for electrodes 6 and 7 are the same. This is because the relative positions of the electrode and eleven charged particles for the two electrodes are the same. Also, it is similar for electrodes 5 and 1. The curves for electrodes 2, 3 and 4 are the same, because the distributions of the charged particles flowing nearby are the same, which would make the most significant contributions to the induced charge.

In addition, the common feature of the eight curves is that the number of induced charges is the largest when the particles are in the position *z* = 0 and decreases with the increasing absolute value of *z*. The difference is that the more charged particles there are near the electrode, the larger the charge induced on it is.

**Figure 6:**
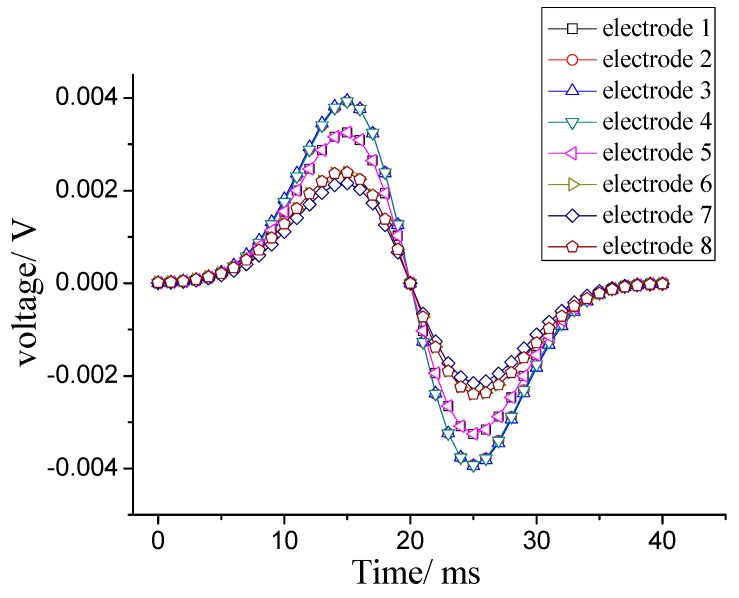
Eight output signals of the ESA.

Thirdly, the current outflow from each electrode is transferred into a voltage signal after passing the interface circuit in the ESA system. The output data *U*(*t*) is expressed as [11]:

(1) $$U(t)=R\frac{dQ(t)}{dt}$$

where *R* is the resistance of the interface circuit. If taking *R* as a constant, the voltage signal is the same as the derivate of the induced charge curves. Figure 6 shows the eight output signals of the eight electrodes. Accordingly, the output signals also have four types. It is worth noting that the greater the charge amount near the electrode, the larger the amplitude value of the output signal from it.

As a consequence of the simulation, only the charged particles flowing near an electrode would make the most significant contributions to its output signal’s amplitude. Thus, the amplitudes of all eight electrodes can give important information on the particles’ distribution in the cross section of the conveying pipe.

### 2.3. ANN

**Figure 7:**
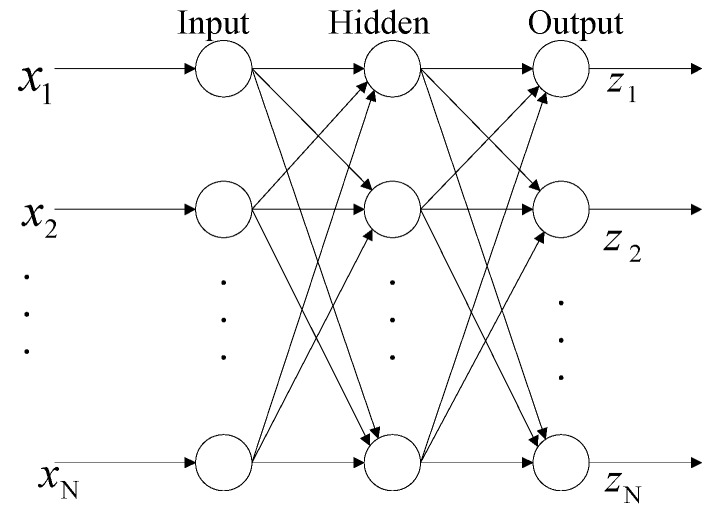
Architecture for three-layer ANN.

ANNs have been developed as an important tool in modeling relations between input and output data [14]. It depends a lot on signal processing elements called neurons. They are associated, and the intensity of the associations is denoted by parameters named weights. There are several kinds of ANN structures. The best known one is the feed forward neural network (FFNN), which is generally divided into multiple layers. Usually, there is an input layer, a few hidden layers, and an output layer. In this work, the FFNN with the network structure shown in Figure 7 was built. The input layer is the first layer that imports all inputs to the network. The second layer is the hidden layers. The output layer is the third layer where outputs are received [15]. The training of the FFNN is done by minimizing the cost function, usually a quadratic function of output error. The most common training algorithm for FFNN is the back propagation (BP) algorithm. Therefore, a BP algorithm was used during the training in the present work.

## 3. Experimental Setup

**Figure 8:**
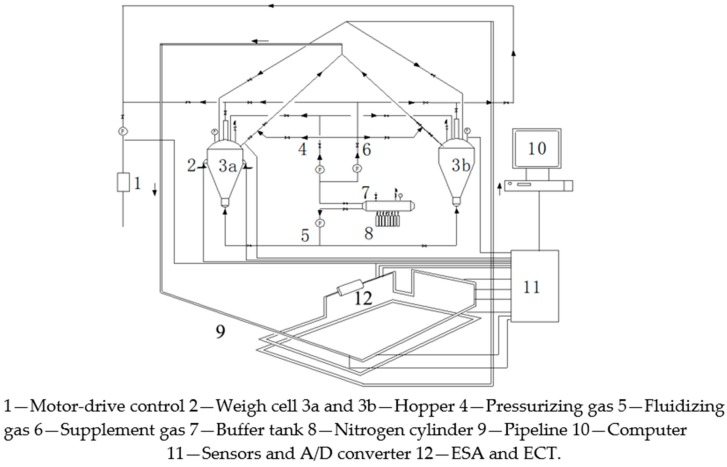
Schematic of a pneumatic conveying system of pulverized coal.

A structure sketch of the pneumatic conveying system of pulverized coal is shown in Figure 8. The experimental setup covers an area of 20 m^2^. Two hoppers with a capacity of 0.648 m^3^ and a height of 1.9 m^2^ adopt the top-discharge and bottom-fluidization arrangement. The pressurizing gas, fluidizing gas and supplement gas are supplied from the buffer tank filled with high-pressure gas. In the feeding hopper, pulverized coal is firstly fluidized by the fluidizing gas, then enters the conveying pipeline, and finally reaches the receiving hopper. The transport steel pipeline has a 10 mm inner diameter and is 45 m long. The ESA and ECT are both installed in position 12 of the conveying pipeline, with a distance of 0.9 m from the bends. The bend radius is 0.06 m. The transported material is pulverized lignite coal, the properties of which are summarized in Table 1.

**Figure 9:**
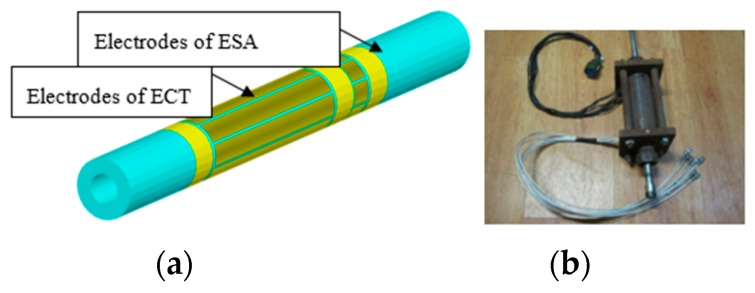
Integrated structures of ESA and ECT (**a**) Integrated electrodes arrangement; (**b**) photograph of the appearance.

The structure of the ECT is much the same as the ESA. The capacitances between each electrode pair are to be detected, which are used to reconstruct the distribution of dielectric materials over the cross section of the vessel or pipe [2,3]. The electrodes of the ECT and ESA are arranged on a quartz glass tube with a length of 200 mm and diameter of 10 mm (shown in Figure 9). In this paper, the ECT system plays a supporting role, which is to reflect the flow patterns of the coal particles in the conveying pipeline, so that the flow pattern would not be observed from outside of the metal pipe. The sampling frequency of the electrostatic signals is 1000 Hz for each channel. The ECT frame rate and resolution of the reconstruction image are 74 frames/s and 32 × 32 pixels, respectively.

## 4. Results and Discussion

### 4.1. Flow Patterns

**Figure 10:**
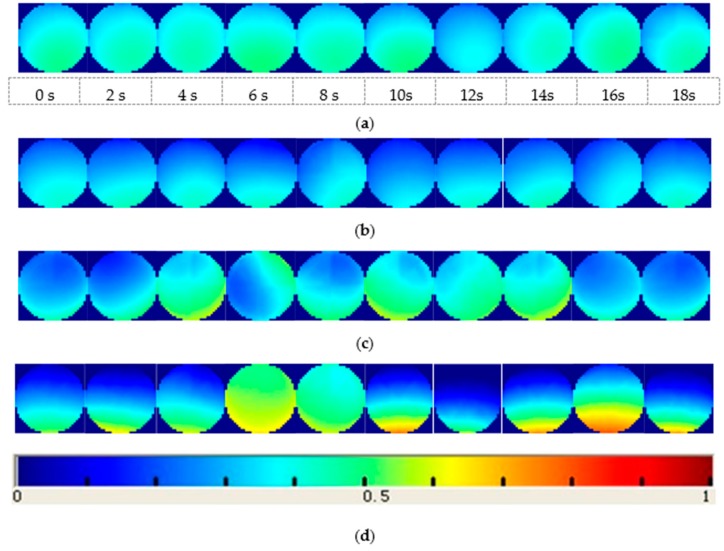
ECT images of four flow patterns (**a**) fully suspended flow; (**b**) stratified flow; (**c**) dune flow; (**d**) slug flow.

In this work, four kinds of gas‒solid two-phase flow patterns are considered: fully suspended flow, stratified flow, dune flow and slug flow. Experimental operating conditions of those flow patterns are shown in Table 2. ECT images (in Figure 10) present particle distributions over the cross section of the pipe of those four patterns respectively (although ECT can be used to identify gas‒solid flow patterns, this paper focuses on an identification method based on an ANN and ESA). The color bar (under the images) ranging from blue to red, indicates the coal particle concentration changes from the minimum to the maximum value. For fully suspended flow presented in Figure 10a, powders are fully suspended, and the transportation process is stable. For stratified flow, as presented as ECT images in Figure 10b, the transportation process is also stable, while the coal particles are not evenly distributed, with one part gathered on the bottom and another part suspended in the upper part of the pipeline. For dune flow, the suspension of the particles is worse than for the fully suspended flow and stratified flow, which is presented as ECT images in Figure 10c. For slug flow, the powders gather and slip along the bottom of the pipeline and powder slug arises intermittently, which is presented in the ECT images in Figure 10d.

### 4.2. System Identification

As shown in Figure 10, the distribution of particles concentration in pipe is nonuniform and nonuniform degree varies with the flow pattern. So, the concentration distribution of particles has a significant relationship with the gas‒solid flow pattern. Based on the analysis in Section 2.2, the amplitudes distribution of the output signals of the ESA reflects the particles distribution in the cross section of the pipeline. Thus, the average value and standard deviation of the eight output signals’ amplitudes were respectively used as the input values of the ANN. Due to the ESA having eight output signals, each set of inputs is an array with eight data, which is expressed as follows:

- the average values of eight output signals’ amplitudes, [*A*_1_, *A*_2_, *A*_3_, *A*_4_, *A*_5_, *A*_6_, *A*_7_, *A*_8_];
- the standard deviation of eight output signals’ amplitudes, [*S*_1_, *S*_2_, *S*_3_, *S*_4_, *S*_5_, *S*_6_, *S*_7_, *S*_8_].

Accordingly, an ANN model was built which has eight input values, one hidden layer and four output values. Four hundred samples were used and divided as follows: 100 for fully suspended flow, 100 for stratified flow, 100 for dune flow and 100 for slug flow. Sixty percent of the data is for training and 40% for testing.

**Figure 11:**
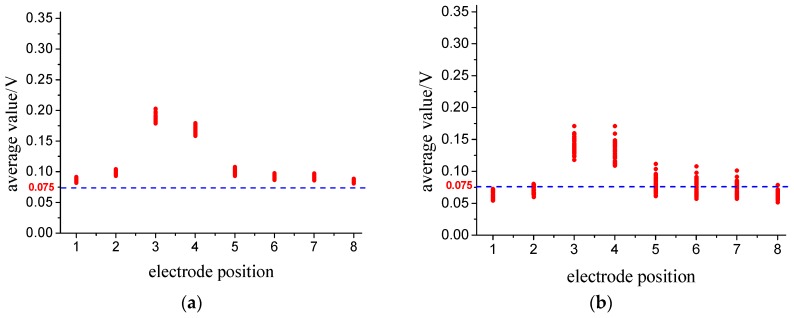

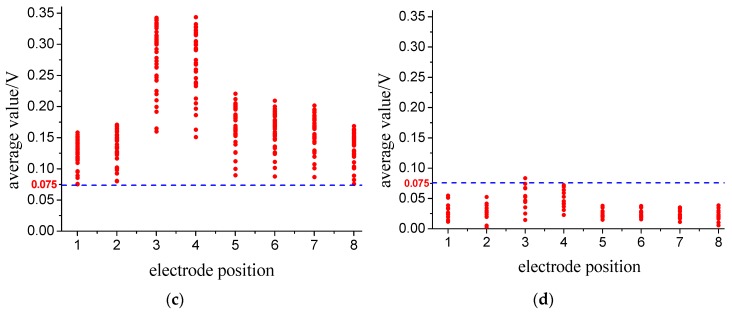
Regions of the four patterns using the average value. (**a**) fully suspended flow; (**b**) stratified flow; (**c**) dune flow; (**d**) slug flow.

**Figure 12:**
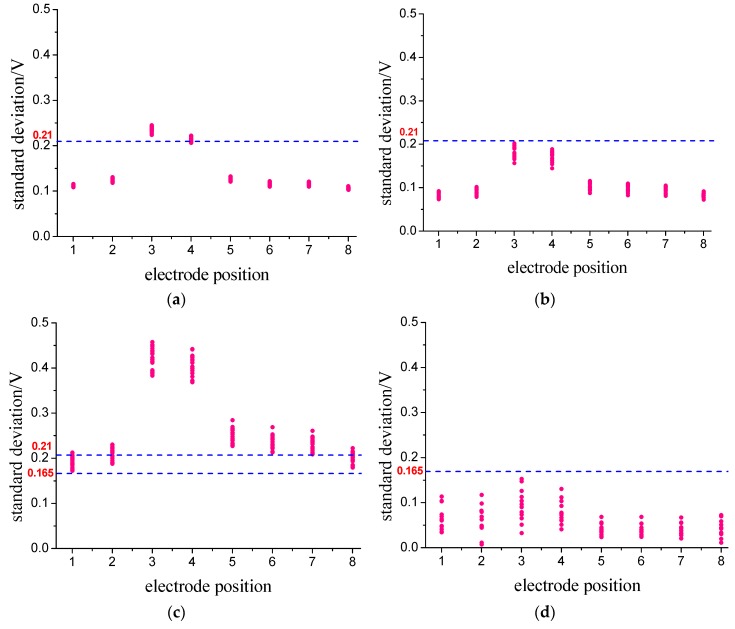
Regions of the four patterns using the standard deviation. (**a**) fully suspended flow; (**b**) stratified flow; (**c**) dune flow; (**d**) slug flow.

Figure 11 shows the average values range of each output signal of the 400 samples for the four flow patterns. It can be seen that: (1) comparing fully suspended flow with stratified flow, there are some overlaps between the regions of *A*_3_, as well as *A*_4_, *A*_5_, *A*_6_, *A*_7_ and *A*_8_. A clear separation of the region for *A*_1_ or *A*_2_ can be observed, indicating that the two flow patterns of fully suspended flow and stratified flow can be differentiated by the value of *A*_1_ or *A*_2_. (2) Between stratified flow and dune flow, there is a clear separation of the region for *A*_1_. Thus, the two flow patterns of stratified flow and dune flow can be differentiated by the value of *A*_1_. (3) Impressively, the overlap disappears between dune flow and slug flow. Figure 12 shows the standard deviation range of each output signal of the 400 samples for the four flow patterns. We can also find the separation regions for one or more *S*_i_ between any two of those four flow patterns.

Table 3 shows the prediction results using the two sets of parameters as the input parameters. A good result was obtained. For four flow patterns, the correct classification rates are 100%.

## 5. Conclusions

To identify flow patterns of gas‒solid two-phase flow in pneumatic conveying pipelines, this paper proposes an identification method based on an ESA and ANN. An ESA can obtain the particles distribution in the conveying pipeline because of its structure. Simulation results have shown that the amplitudes of output signals from eight electrodes can give important information on the particles distribution and further infer the flow patterns of the gas‒solid flow. Based on that, the average value and standard deviation of the output signals’ amplitude are respectively taken as the inputs of the ANN to identify fully suspended flow, stratified flow, dune flow and slug flow in experiments. Results show that for any one of those two input values, the correct recognition rates of the ANN model are 100%. The method of utilizing an ESA and ANN is not restricted by materials and conveying parameters, as long as there is variation of flow patterns and particle charging.

## Figures and Tables

**Table 1:** Properties of lignite coal particles.

Material	Mean Size/μm	Density/kg·m^−3^	Resistivity/(Ω·m)	Moisture Content/%
Lignite	208.5	1350	2.3 × 10^12^	10.39

**Table 2:** Experimental operating conditions of the four flow patterns.

Flow Pattern	Total Transportation Differential Pressure/MPa	Carrier Gas	Solid Mass Flow Rate/kg·s	Gas Velocity/m·s^−1^	Ratio of Solid‒Gas Mass
Fully suspended flow	1.00	CO_2_	0.264	8.284	5.8
Stratified flow	0.75	CO_2_	0.218	6.815	6.2
Dune flow	0.50	N_2_	0.192	4.757	15.7
Slug flow	0.33	N_2_	0.128	4.029	12.8

**Table 3:** Percent of correct classification.

	Correct Classification			
Parameter/Pattern	SF	LF	DDF	DF
[*A*_1_, *A*_2_, *A*_3_, *A*_4_, *A*_5_, *A*_6_, *A*_7_, *A*_8_]	100%	100%	100%	100%
[*S*_1_, *S*_2_, *S*_3_, *S*_4_, *S*_5_, *S*_6_, *S*_7_, *S*_8_]	100%	100%	100%	100%
